# Wastewaters treatment containing phenol and ammonium using aerobic submerged membrane bioreactor

**DOI:** 10.1186/s13065-018-0450-1

**Published:** 2018-07-09

**Authors:** Mashallah Rezakazemi, Mohsen Maghami, Toraj Mohammadi

**Affiliations:** 1 Faculty of Chemical and Materials Engineering, Shahrood University of Technology, Shahrood, Iran; 2 Research and Technology Centre for Membrane Processes, Faculty of Chemical Engineering, Iran University of Science and Technology (IUST), Narmak, Tehran, Iran

**Keywords:** Membrane, Hollow fiber, Wastewater, Ammonium removal, Environment

## Abstract

Phenolic wastewater was treated using anaerobic submerged membrane bioreactor (ASMBR). Effect of different solids retention times on MBR performance was studied. Various ratios of carbon to nitrogen were used in the synthetic wastewaters. During the operation, phenol concentration of feed was changed from 100 to 1000 mg L^−1^. Phenol concentration was increased stepwise over the first 30 days and kept constant at 1000 mg L^−1^, thereafter. For the first 100 days, a chemical oxygen demand (COD) to N ratio of 100:5.0 was used and this resulted in phenol and COD removal more than 99 and 95%, respectively. However, the ammonium removal decreased from 95 to 40% by increasing the phenol concentration of feed, from 100 to 1000 mg L^−1^. For the last 25 days, a COD to N ratio of 100:2.1 was used due to the ammonium accumulation in the ASMBR. This led to the complete ammonium removal and no ammonium was detected in the ASMBR permeate. These results suggest that in the ASMBR at high phenol loading of 1000 mg L^−1^, COD to N ratio of the phenolic wastewater must be 100:2.1 for ammonium removal, while at low phenol loading, COD to N ratio of 100:5.0 can be used.

## Introduction

Many industrial wastewaters are produced yearly in the world [1, 2]. Most of them contain ammonia [3, 4], phenolic and nitrogenous compounds at high concentrations, e.g. wastewaters from coke industries and refineries [5, 6]. Treatment methods such as liquid–liquid extraction [7], ultrasound [8], adsorption [9], membrane processes [10] and biological treatment methods have been used for phenolic wastewater renovation. The membrane separation processes can be used in the different process including gas separation [11–20], pervaporation [21–27], filtration in different configuration particularly hollow fiber membrane contactor [28–33]. Among these, biodegradation seems to be a suitable treatment method for phenol and ammonium removal due to the neutral compounds produced in this process [34]. But, in biological systems, phenol has a negative effect on nitrogen removal [35]. Previous studies have shown that real and synthetic phenolic wastewaters can be treated by biological systems [34]. Effect of phenol concentration on nitrification process in batch systems and activated sludge reactors was investigated by Amor et al. [34]. The results showed that the extent of nitrification decreases with initial phenol concentration above 1000 mg L^−1^, and also, increasing initial phenol concentration causes reduction of the nitrification along with the accumulation of nitrite and ammonium. Coke industries use activated sludge for wastewater treatment and revealed an inhibitory effect of phenol and *p*-cresol on nitrification. The results showed that no nitrification occurs at phenol concentrations above 500 mg L^−l^. In addition, effects of initial concentrations of ammonium, thiocyanate, free cyanide, ferric cyanide on nitrification were studied by [36]. Other researchers also treated wastewaters of coke industries by conventional activated sludge process at several conditions and showed that phenol removal is more than 98% while ammonium removal is very low [37]. Effects of phenol and formaldehyde on denitrification at anoxic conditions were studied and the results showed that removal of nitrogen decreases with increasing phenol [38].

The membrane bioreactor (MBR) is a technology which can be used in phenolic wastewater treatment [39]. Different cultures were used as biological systems to remove phenol. *Pseudomonas putida* [40] and activated sludge [41] are usually used as a biological system in MBRs and the results showed suitable phenol biodegradation in both systems. Biodegradation of high loading phenol in MBRs was also studied and a population of biological systems was investigated [42]. The results showed that floating biodegradative populations are observed and they may inhibit high efficiency and stability of the treatment performance. Researches showed that activated sludge must be acclimatized in presence of phenol to be used in MBR [43]. Although the removal of phenol in membrane bioreactor has been studied, phenol to nutrients ratio (especially nitrogen) in feeds has not been investigated.

Membrane bioreactor performance also affected by many factors, i.e. hydraulic residence time (HRT), sludge retention time (SRT), temperature, feed to microorganism ratio (F/M), mixed liquor suspended solids (MLSS), aeration (as oxygen source and membrane fouling reducer) and biomass properties. The effects of these parameters on MBR performance and membrane fouling have been the subjects of some studies [44]. Among these factors, HRT is one of the most influencing factors since it is directly related to the reactor volume and this, in turn, affects the capital and operational costs [45]. Using ceramic membranes in external MBRs for phenol removal showed their ability to operate at low HRTs and high loadings of phenol [40]. Real wastewater of a refinery containing phenol and ammonium was also treated by MBR and no phenol was detected in effluent but the effect of phenol on ammonium removal was not studied [35].

In this study, MBR has operated at different chemical oxygen demand (COD) to N ratios with phenol as a carbon source and three SRTs (infinite, 30, and 10 days) to evaluate ammonium accumulation inside the bioreactor.

## Materials and methods

### Activated sludge and synthetic wastewater

Activated sludge used in this study was supplied from Tehran oil refinery. A synthetic phenolic wastewater was prepared based on the mineral medium as described by Ahn et al. [41]. Mineral medium contained 0.067 g CaCl_2_·2H_2_O, 0.248 g MgCl_2_·6H_2_O, 0.5 mg FeSO_4_·7H_2_O, 0.4 mg ZnSO_4_·7H_2_O, 0.002 mg MnCl_2_·4H_2_O, 0.05 mg COCl_2_·6H_2_O, 0.01 mg NiCl_2_·6H_2_O, 0.015 mg H_3_BO_3_, and 0.25 mg EDTA per liter. To this medium, NH_4_Cl as a nitrogen source as well as KH_2_PO_4_ and K_2_HPO_4_ as phosphorus sources were added at desired amounts. Glucose and/or phenol were used as carbon source and phenol at various concentrations.

### SMBR setup

**Fig. 1:**
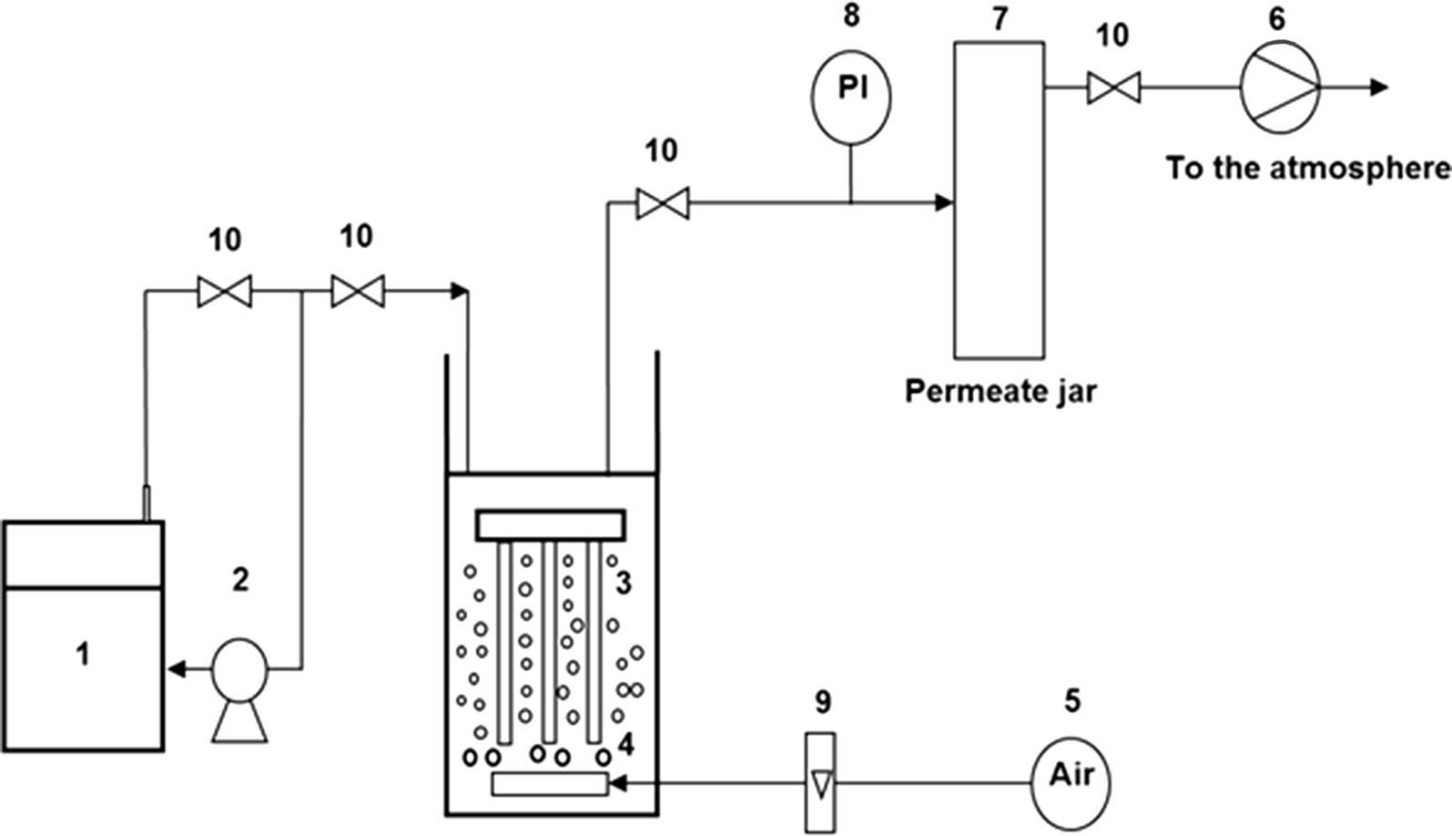
Schematic drawing of the ASMBR; 1—feed tank 2—feed pump 3—U type membrane module 4—air diffuser 5—air pump 6—vacuum pump 7—permeate tank 8—pressure sensor 9—air flow meter 10—valves

The SMBR used in this study consisted of a glass reactor with a working volume of 10 L (Fig. 1) and an immersed polyvinylidene fluoride (PVDF) hollow fiber membrane module with a surface area of 5 × 10^−2^ m^2^ and pore size of 0.5 µm. The length of microfiltration module was 0.4 m. Synthetic wastewater kept in a steel tank was fed to the bioreactor by a peristaltic pump at desired flow rates. The effluent was removed through the membrane and collected in a permeate tank via a vacuum pump. Vacuum pressure was measured by a pressure sensor and controlled accordingly. The reactor was aerated using an air pump and several porous diffusers. Steel-made junctions were used where necessary to prevent oxidation. To monitor pH and DO, a pH meter (Lab-215, palintest Inc.) and a DO probe (HACH, Germany) were also installed in the ASMBR.

### Experiments

The bioreactor was filled with activated sludge at initially MLSS of 3500 mg L^−1^. The SMBR was operated at 23–24 °C in a continuous flow mode throughout this study by introducing 10 mL min^−1^ feed to bioreactor resulting in an HRT of 16.6 h. Aeration was carried out such that DO remains above 2 mg L^−1^. Constant permeate flux was maintained using air backwashing during SMBR operation. Chemical cleaning was used when TMP reached 0.35–0.40 bar to completely remove the accumulated biomass from membrane skein and this allowed operation at TMPs of 0.05–0.10 bar.

Six experiments were carried out within 125 days (Table 1). In run 1, initially feed at COD of 300 using glucose as sole carbon source and COD:N:P ratio of 100:5:1 was used. This was followed by gradual substitution of glucose with phenol at constant COD of 300 mg L^−1^ within days 5 and 10 for acclimatization of activated sludge to phenol. Subsequently, phenol concentration in the feed was increased stepwise up to 500 mg L^−1^, resulting in an influent COD of 1190 mg L^−1^ at the end of run 1 at day 30. Phenol concentration was further raised to 1000 mg L^−1^ in run 2 and remained constant thereafter. Runs 3, 4 and 5 were performed to investigate the effect of SRTs at 30, 10 and infinity (days), respectively. Variation of SRTs was accomplished by discharging sludge in continuation of run 5, COD:N:P ratio was changed to 100:2.1:0.4 to examine its effect on MBR performance. This was followed by reducing SRT to 10 days in run 6 to test the effect of SRT at COD:N ratio of 50.

**Table 1:** ASMBR operational parameters

No. interval	Operation interval (day)	SRT (day)	Phenol (mg L^−1^)	COD: N-NH_4_^+^	MLSS (mg L^−1^)
RUN (1)	30	∞	0–500	20	3500–5000
RUN (2)	30	∞	1000	20	5000–8500
RUN (3)	17	30	1000	20	5000–7200
RUN (4)	14	10	1000	20	5000–6000
RUN (5)	20	∞	1000	20–50	5000–8500
RUN (6)	14	10	1000	50	5000–6000

### Analytical methods

Chemical oxygen demand and MLSS were measured according to the standard methods (ASTM D1426 for Ammonia Nitrogen In Water; ASTM D1783 for Phenolic Compounds in Water; Greenberg 5520 B for COD; Greenberg 2540 D for TSS). Phenol, N-NH^4+,^ and COD were measured by spectrophotometry. COD was measured after centrifuging the mixed liquor for 10 min at 3000*g*. For measuring MLSS, three samples were taken each time. The samples were filtered through a 0.45 µm Millipore filter and dried in an oven at 105 °C until obtaining constant weights. The average values were then calculated.

## Results and discussion

### SMBR performance during acclimatization of activated sludge

**Fig. 2:**
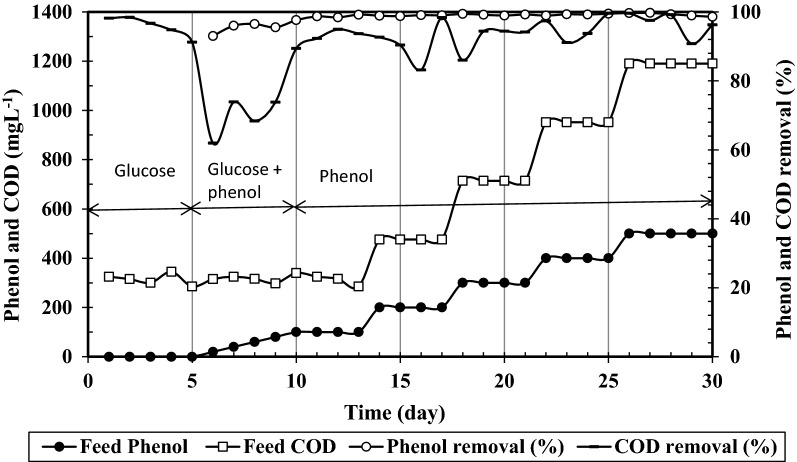
Phenol and COD concentrations in feed and their removal efficiencies during acclimation of activated sludge (Run 1)

**Fig. 3:**
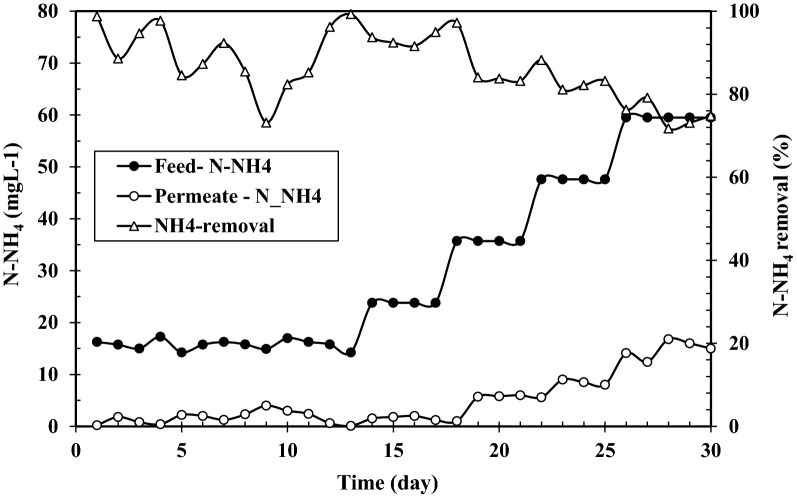
N-NH_4_^+^ concentration in feed and permeate and N-NH_4_^+^ removal during acclimation of activated sludge (Run 1)

SMBR performance during acclimation of activated sludge (Run 1) is shown in Figs. 2 and 3. For the first 5 days, influent containing glucose as sole carbon source at COD of 300 mg L^−1^ and COD:N:P ratio of 100:5:1, a common ratio used in aerobic activated sludge processes, was exploited to increase the MLSS of activated sludge in SMBR where more than 96% COD removal was obtained. Due to the toxicity of phenol, acclimation of activated sludge was then carried by gradual substitution of glucose by phenol at constant influent COD during days 5–10. A sharp decrease can be seen in COD removal by introducing phenol to SMBR. However, due to the adaptation of activated sludge, COD removal raises again up to 90% at day 10. For further adaption to higher phenol concentrations, influent COD was increased stepwise up to about 1200 mg L^−1^ by increasing phenol concentration up to 500 mg L^−1^ until day 30. COD and phenol removals during this stage remained over 85 and 95% and finally reached about 95 and 99%, respectively. These results confirmed that the activated sludge was suitably acclimated with phenol such that microorganisms could completely utilize phenol at up to 500 mg L^−1^ as a sole carbon source, with an insignificant effect on phenol removal [46].

Ammonium concentration in influent and permeate as well as ammonium removal during acclimation period are presented in Fig. 3. A constant COD:N ratio of 100:5 was used to provide enough carbon and nitrogen for growth of the microorganisms [47]. Within the 1st days, when glucose was used as carbon source, almost all N-NH4+ was removed and nitrogen detected in the effluent was below 5%. To maintain a constant COD:N ratio while influent phenol concentration was raised, influent ammonium concentration was accordingly raised (Fig. 3). By introducing phenol to SMBR, ammonium removal started to decrease however after 10 days it increased showing similar trends to COD removal in Fig. 2.

By increasing influent phenol concentration to higher than 400 mg L^−1^, ammonium removal dropped to about 80% and further decreased to 75% at 500 mg L^−1^ influent phenol. This attributed to the fact that phenol reduced nitrification and excess ammonium did not be removed.

### Effect of sudden phenol loading on SMBR performance

**Fig. 4:**
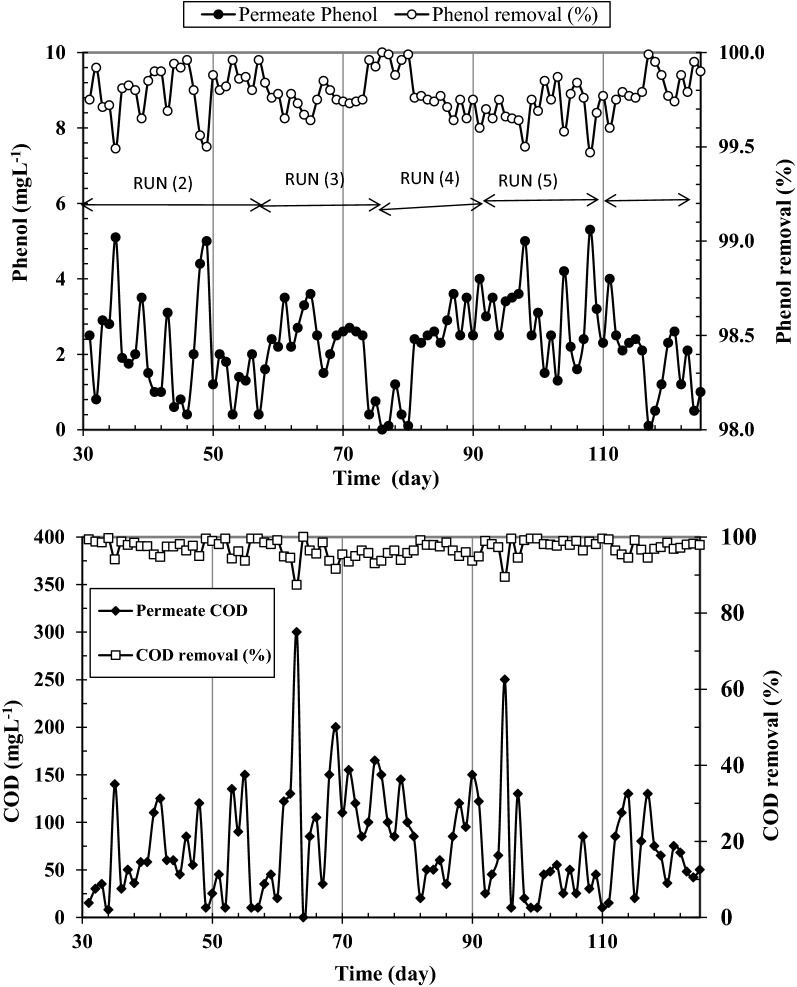
Permeate COD and phenol concentrations and removal efficiencies at influent phenol concentration of 1000 mg L^−1^

**Fig. 5:**
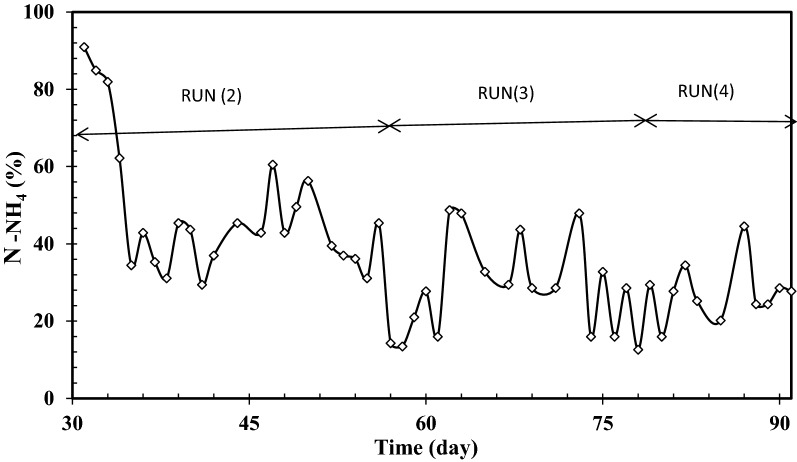
N-NH_4_^+^ removal at SRTs of infinity, 30 and 10 days, influent phenol concentration of 1000 mg L^−1^ and COD:N ratio of 100:5

To investigate how SMBR responds to a change in phenol loading, influent phenol concentration was increased to 1000 mg L^−1^ (COD of about 2400 mg L^−1^) at constant COD:N ratio in run 2. This concentration of phenol was reported to be toxic to microorganisms [41]. Results given on the left side of Figs. 4 and 5 show that almost all phenol was biodegraded after 2 days. Phenol and COD removals of higher than 98 and 95% are evidence for the complete mineralization of phenol with no organic intermediate materials in SMBR, which is favorable for phenolic wastewater treatment. The results are similar to those obtained by other researchers [41].

Despite the efficient COD and phenol removals observed at 1000 mg L^−1^ influent phenol in run 2 which are similar to those observed in acclimation period (Fig. 2), ammonium removal decreased sharply and leveled off after 6 days. Overall it can be seen that by increasing phenol concentration from around 300–500 (Fig. 2) and further to 1000 mg L^−1^ (Fig. 5), ammonium removal decreases from around 90–75 and 40%, respectively. This means that high loading of phenol adversely affects ammonium removal. This phenomenon has been reported to occur by some other researchers for conventional activated sludge processes [48] and it has mostly been attributed to inhibition of nitrifying bacteria converting ammonium ions to nitrate.

### Effect of sludge retention time (SRT) on SMBR performance

Having increased the influent phenol concentration to 1000 mg L^−1^ in run 2 at infinite SRT (no sludge discharge), two further experiments were performed at SRTs of 30 and 10 days in runs 3 and 4 to examine the effect of SRT on SMBR performance at COD:N ratio of 20. Phenol and COD concentrations in permeate and their removals at the three examined SRTs are shown in Fig. 4.

As can be seen in Fig. 4, reduction of SRT had an insignificant effect on phenol and COD removals. Comparison of the results showed that at the three examined SRTs more than 99.5% of influent phenol and 95% of influent COD were removed. With regards to ammonium removal, Fig. 5 shows that by reducing SRT from infinity to 10 days ammonium removal undergoes a decrease from 40 to around 25%.

### Effect of COD:N ratio on SMBR performance

**Fig. 6:**
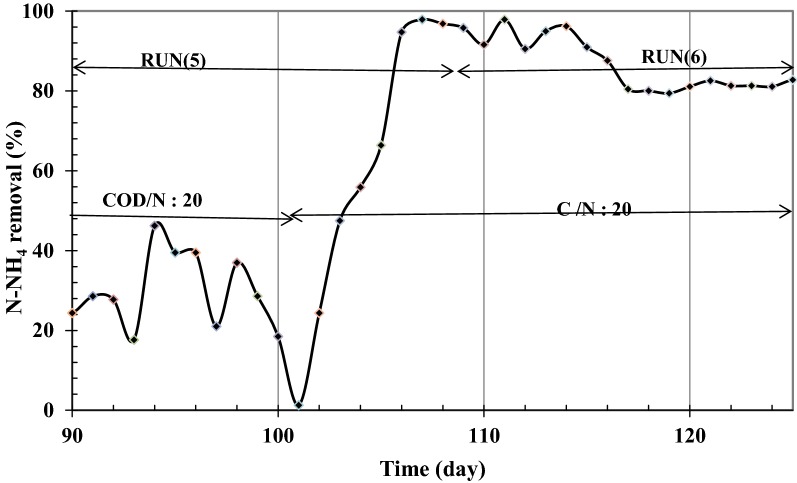
N-NH_4_^+^ removal for 1000 mg L^−1^ phenol in feed for COD:N ratio of 100:2.1 and SRT of infinite and 10 days

In runs 1 and 2, it was observed that by increasing influent phenol concentration from 300 to 1000 mg L^−1^ at constant COD:N ratio of 20 ammonium removal decreased from about 90–40% while no significant changes were observed in COD and phenol removals such that they remained above 95 and 99.5%, respectively. This means that nearly all influent COD is consumed and hence small amounts of phenol and COD remains in SMBR. In contrast, ammonium concentration in SMBR increased with influent COD concentration. Estimation of the consumed COD:N ratios at various examined influent concentrations given in Table 2 shows that the consumed ratios are all higher than the influent COD:N ratio of 20 in run 1 and in run 2 this ratio reaches nearly 52. To check whether this phenomenon occurs due to high phenol (COD) or ammonium concentration, in run 5 influent ammonium concentration was decreased at constant phenol concentration of 1000 mg L^−1^ such that an influent COD:N ratio of around 50 was obtained (Fig. 6). The high value of ammonium removal in addition to COD removal shows that at each phenol (COD) loading rate a unique COD:N ratio is required and a constant influent COD:N ratio.

**Table 2:** COD to N ratios in influent and effluent of SMBR (phenol as carbon source)

Influent				Consumed	
COD:N ratio	COD (mg L^−1^)	Phenol (mg L^−1^)	NH_4_ (mg L^−1^)	NH_4_ (mg L^−1^)	COD/NH_4_ ratio
20	250	100	15	12	20.8
20	450	200	22.5	19.5	23.1
20	700	300	35	28	25
20	950	400	47	37	25.7
20	1190	500	59	43	27.7
20	2380	1000	118	46	51.7
50	2380	1000	48	45	52.9

To investigate the effect of SRT on ammonium accumulation in SMBR at COD:N ratio of 50, run 6 was performed at SRT of 10 days. Results in Fig. 4 showed that COD and phenol removals remain above 95 and 99%, respectively. However, ammonium removal rapidly grows such that ammonium removal reaches more than 90% after 4 days as shown in Fig. 6. The lower ammonium removal at SRT of 10 days can be attributed to the un-complete bacterial accrue in SMBR.

### Transmembrane pressure (TMP)

**Fig. 7:**
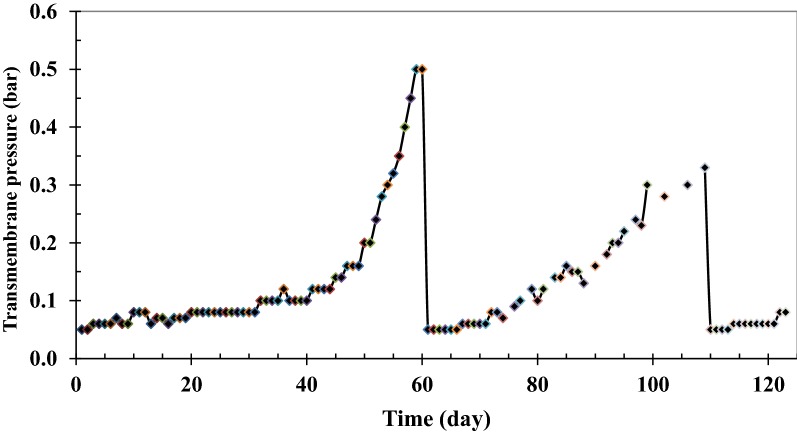
TMP and membrane permeability throughout the SMBR operation period

To run the SMBR in continuous mode, the synthetic wastewater was fed to the reactor with the desired flow rate (12 L/m^2^.h) and the effluent was removed with the same flow rate. During the experiments, blocking materials move towards the membrane surface. The membrane then absorbs these materials, hence, fouling occurs, and generally, TMP increased for maintaining a constant permeate flux. To remove reversible fouling during operation, therefore, air backwashing and washing with distilled water was performed and by reaching a TMP of 0.35–0.40 bar, the membrane was chemically cleaned. TMP profile during the six experiments is presented in Fig. 7.

As can be seen in Fig. 7, TMP was increased due to the reduction in membrane permeability. After 60 days, the membrane was severely fouled and chemical cleaning was used to remove the fouling. NaOH (0.4 wt%) was used to remove the organic materials and HCl (1%) was used for inorganic materials. The membrane was immersed in these solutions for 1 h and 30 min, respectively, according to the membrane manual. As observed, all of the fouling was removed by this method and the cleaned membrane operated similarly to the fresh membrane.

## Conclusion

In this study, an ASMBR was operated for 125 days to treat a synthetic phenolic wastewater. The ASMBR was operated in 6 runs to investigate the effect of SRT and influent COD:N ratio at 1000 mg L^−1^ influent phenol on bioreactor performance. The following conclusions can be drawn from the present study: (I) It was demonstrated that different concentrations of phenol could be removed by the ASMBR confirming the high capability of the ASMBR in phenol removal. (II) Increasing ammonium as nitrogen source caused ammonium accumulation at phenol concentrations above 500 mg L^−1^, however, it had no effect on phenol and COD removals and COD to N ratio of 100:2.1, was capable of removing ammonium accumulation. (III) Using air backwashing for cleaning of the hollow fiber membrane module fairly reduced fouling and the membrane could be operated for 60 days without chemical cleaning. Chemical cleaning was then performed effectively.
